# Finding Peace in Pixels: Exploring the Therapeutic Mechanisms of Virtual Nature for Young Adults’ Mental Well-Being

**DOI:** 10.3390/healthcare13080895

**Published:** 2025-04-14

**Authors:** Ka-Po Wong, Sikai Wu, Haoneng Lin, Kean Poon, Bohan Zhang, Jing Qin

**Affiliations:** 1 Department of Applied Social Sciences, The Hong Kong Polytechnic University, Hong Kong, China; 2 Centre for Smart Health, School of Nursing, The Hong Kong Polytechnic University, Hong Kong, China; sikai.wu@connect.polyu.hk (S.W.); exusiai.lin@polyu.edu.hk (H.L.); bohan.zhang@connect.polyu.hk (B.Z.); harry.qin@polyu.edu.hk (J.Q.); 3 School of Education, The University of New South Wales, Kensington, NSW 2052, Australia; kean.poon@unsw.edu.au

**Keywords:** virtual reality, nature exposure, mental health, stress reduction, young adults, thematic analysis

## Abstract

**Background:** This investigation examines the phenomenological dimensions of young adults’ engagement with virtual natural environments for psychological stress amelioration through rigorous thematic analysis. Contemporary epidemiological data reveal a concerning prevalence of stress among young adults aged 18 to 29 years, with approximately 30% reporting moderate to severe manifestations. Despite virtual reality (VR)’s emergence as a promising modality for mental well-being interventions, a significant lacuna exists regarding the qualitative understanding of these immersive experiences. **Methods**: Through semi-structured interviews with 35 young adults following a four-week VR nature intervention, we constructed a conceptual framework comprising five interconnected strata: intervention, experience, process, context, and outcome. **Results**: Our analysis illuminated intricate bidirectional relationships among sensory elements, emotional responses, immersion depth, interactive affordances, post-session effects, psychological development, implementation challenges, individual variability, and comparative efficacy. The findings demonstrate congruence with both Attention Restoration Theory and Stress Recovery Theory while necessitating consideration of technology-specific mediators. Notably, the identified “stress barrier” phenomenon temporarily inhibited intrusive cognitions, suggesting promising therapeutic mechanisms. Pronounced heterogeneity in environmental preferences and psychophysiological responsiveness underscores the imperative for personalized implementation strategies. **Conclusions**: These insights provide substantive guidance for VR nature applications across therapeutic, occupational, and educational domains, potentially augmenting our repertoire for addressing stress-related sequelae in contemporary society.

## 1. Introduction

Contemporary epidemiological data reveal an alarming trajectory in the mental health landscape among young adults aged between 18 and 29, with stress emerging as a predominant concern of considerable magnitude [1]. Recent statistics indicate that approximately 30% of this demographic in Hong Kong report experiencing moderate to severe stress levels [2], underscoring the escalating public health implications of this phenomenon [3]. The World Health Organization defines stress as a state of psychological tension triggered by challenging circumstances [4]. Although stress represents a normative physiological response that enables individuals to address threats and challenges, the efficacy of one’s coping mechanisms substantially influences overall wellbeing. This consideration is particularly salient for emerging adults navigating the complex developmental tasks characteristic of this transitional life stage.

The developmental period spanning 19 to 29 represents a heightened vulnerability window for mental health disorder onset [2], as individuals experience significant life transitions that frequently precipitate psychological distress [5]. Young adults encounter distinct psychosocial stressors that may compromise psychological health and potentially engender long-term health impacts [6]. These stressors include academic performance expectations, career establishment challenges, financial precarity, and social media-related pressures [7,8].

Conventional approaches to stress management and relaxation for this population have historically included various modalities, such as mindfulness meditation, progressive muscle relaxation, cognitive-behavioral techniques, and physical exercise regimens [9]. While these interventions demonstrate empirical efficacy, they frequently present substantial barriers to implementation, including significant time investments, specialized training requirements, and accessibility limitations [10]. Moreover, young adults consistently report difficulties maintaining adherence to these practices amid competing priorities and obligations. As numerous scholars have observed, the intricate complexities of contemporary existence may induce psychological distress even among individuals without pre-existing mental health conditions [11,12].

Exposure to natural environments has long been acknowledged for its restorative properties and stress-ameliorating effects [13]. However, accelerating urbanization, temporal constraints, and geographical limitations increasingly restrict access to natural settings, particularly for young adults residing in metropolitan areas [14]. This growing disconnection from nature has catalyzed the exploration of technological alternatives that might effectively simulate the beneficial aspects of nature exposure.

Virtual reality (VR) technology has emerged as a promising modality for mental wellbeing interventions, offering immersive, controlled environments accessible regardless of physical location or temporal constraints [15]. Preliminary quantitative investigations suggest that VR-based natural environments may effectively reduce physiological markers of stress and subjective anxiety levels [16,17]. This finding assumes particular significance given that anxiety disorders constitute the most prevalent category of mental health conditions globally [18], with an estimated 4.05% of the global population (301 million people) experiencing anxiety disorders, and alarmingly, the number of affected individuals has increased by more than 55% from 1990 to 2019 [19].

Despite these promising quantitative indicators, a significant lacuna exists in the literature regarding the qualitative dimensions of these experiences—specifically, how young adults perceive, interact with, and derive therapeutic benefits from virtual natural environments. This methodological gap is particularly problematic as it limits our understanding of the complex psychosocial mechanisms through which VR nature interventions exert their effects. Without this phenomenological insight, practitioners face considerable challenges in optimizing such interventions for maximal therapeutic efficacy and addressing implementation barriers. As numerous studies have documented, young adults experience elevated anxiety levels throughout their daily functioning [20,21], with prevalence rates reaching 50% in university populations [22]. This vulnerable period coincides with significant life transitions, identity formation processes, and neurobiological development, creating a perfect storm of psychological vulnerability [23,24]. Traditional therapeutic approaches often face substantial barriers among this population, including stigma concerns, accessibility limitations, and engagement challenges [25].

The present investigation addresses this research gap by exploring the phenomenological experiences of young adults engaging with VR natural environments for relaxation purposes. Through rigorous thematic analysis of participant interviews, this study addresses four principal research questions:

1. How do young adults experience VR-based natural environments designed for relaxation purposes?
2. What elements of these virtual environments most substantially contribute to or detract from relaxation outcomes?
3. How do participants perceive the comparative effectiveness of VR nature exposure relative to alternative relaxation techniques?
4. What implications do these experiences suggest for the design and implementation of VR-based wellbeing interventions?

By elucidating these qualitative dimensions, this research aims to provide insights into the lived experiences of young adults engaging with VR nature environments, deepening our understanding of their mental wellbeing implications. VR nature experiences show promise in addressing psychological needs while overcoming traditional barriers to intervention. This demographic’s documented receptivity to technology [26] and susceptibility to immersion [27] is particularly relevant given how modern demands have transformed the young adult experience [28,29]. Qualitative inquiry reveals nuanced implementation factors, user experiences, and therapeutic mechanisms that quantitative approaches might overlook. The findings can guide technology developers and mental health practitioners in creating effective VR interventions tailored to young adults’ psychological needs and preferences.

### 1.1. Theoretical Framework

This investigation is anchored in two complementary theoretical paradigms that provide robust explanatory mechanisms for understanding how natural environments—including their virtual simulations—may promote relaxation and enhance psychological wellbeing: Attention Restoration Theory (ART) and Stress Recovery Theory (SRT).

#### 1.1.1. Attention Restoration Theory (ART)

Attention Restoration Theory, initially conceptualized by Kaplan and Kaplan [30] and subsequently refined by Kaplan [31], offers a sophisticated cognitive framework for comprehending the restorative effects of natural environments. ART proposes that directed attention resources become depleted through sustained mental effort, leading to fatigue symptoms including reduced concentration and increased stress [32]. Natural environments restore these cognitive resources through four key components [33]: “being away” (psychological distance from stressors), “fascination” (effortless attention engagement), “extent” (immersive environmental coherence), and “compatibility” (alignment with psychological needs) [31]. VR nature simulations potentially replicate these restorative mechanisms by creating psychological separation from real-world contexts [34], engaging effortless attention [35], generating immersion through multisensory stimuli [36], and aligning with users’ relaxation preferences [37]. ART provides a framework for interpreting our themes of “immersion and disconnection” and “sensory elements”, suggesting VR nature experiences may promote relaxation through cognitive mechanisms paralleling physical nature exposure.

#### 1.1.2. Stress Recovery Theory (SRT)

Complementing the cognitive emphasis of ART, SRT [38,39] addresses the psychophysiological dimensions of nature’s restorative effects, proposing that exposure to natural environments triggers immediate emotional responses that initiate positive physiological changes, including reduced sympathetic nervous system activity and enhanced parasympathetic activation [40]. SRT attributes these responses to evolutionary adaptations [41], suggesting specific natural elements—water features, moderate complexity, spatial depth, and vegetation—signal safety and resource availability, promoting stress reduction through phylogenetically prepared pathways [42]. This framework contextualizes our themes of “emotional impact” and “post-intervention effects”, suggesting VR environments may activate similar unconscious responses as actual nature exposure when they incorporate elements that evolutionarily signal safety and resource abundance [43].

The integration of these theoretical frameworks—ART addressing cognitive restoration processes and SRT addressing emotional and physiological recovery mechanisms—provides a comprehensive foundation for understanding how VR natural environments might promote relaxation and stress reduction among young adults. This dual theoretical framework acknowledges both the attentional and emotional pathways through which virtual nature may exert its beneficial effects, informing both the interpretation of our empirical findings and future design considerations for VR wellbeing applications.

## 2. Methodology

### 2.1. Study Design

A qualitative research method was chosen for this study to explore the phenomenological dimensions of young adults’ engagement with virtual natural environments for stress reduction. Qualitative approaches allow researchers to gain an in-depth understanding of human experiences in personal and social contexts [44], providing insights into the factors that contribute to participants’ experiences [45].

### 2.2. Participant Recruitment

Participants were recruited through purposive sampling using university notice boards, social media announcements, and referrals. Inclusion criteria required participants to be young adults between 18 and 29 years of age [2] with no history of photosensitive epilepsy, severe motion sickness, or significant visual impairments that would interfere with VR use. We continued interviewing participants until reaching data saturation, the point where conversations began yielding repetitive information with no new categories or insights emerging. This approach ensured comprehensive thematic coverage while adhering to established qualitative research practices [46].

### 2.3. Ethical Considerations

All participants provided informed consent before participation, and the study received approval from the University Ethics Committee (approval code: HSEARS20230429001).

### 2.4. Intervention

The intervention was a four-week design, with participants engaging in four individual VR nature experiences in a uniform sequence. Each session lasted 15 min and was scheduled weekly, allowing for consistent and spaced exposure to virtual nature environments. All participants experienced the environment in the same predetermined order: undersea, sky, forest, and river. Participants were not given a choice of which environment to experience during each session.

The VR-based intervention comprised four distinct nature environments, each accompanied by carefully curated musical compositions featuring soft, joyful melodies that harmonize with the visual landscapes. Participants experienced natural environments using Oculus Quest 2 headsets. The Oculus Quest 2 headsets used in this study featured a resolution of 1832 × 1920 pixels per eye, with a refresh rate of 72 Hz. All VR content was rendered at the native resolution and frame rate of the device. The 360-degree video environments were captured at 5.7 K resolution (5760 × 2880) using a professional-grade 360-degree camera and processed to maintain consistent brightness and color accuracy across all sessions. The standalone headsets eliminated the need for external computers, enhancing portability while maintaining adequate visual fidelity. Within each environment, participants could freely look around in 360 degrees to observe the surroundings but could not interact with or manipulate elements within the virtual world. The passive observation design was intentional to ensure consistent exposure across participants. Supplementary Materials File S1 provides example screenshots of the four scenarios.

- Undersea: A marine ecosystem characterized by intricate coral reefs and a diverse array of marine species, including vibrant fish and playful dolphins. The environment featured immersive underwater acoustics that enhanced the sensory experience.
- Sky: An expansive aerial vista presenting a dynamic landscape with nuanced chromatic transitions of sunrise and sunset. The panoramic view integrates urban architectural elements, mountainous terrain, and atmospheric phenomena, including drifting clouds and subtle environmental gradients.
- Forest: An ecological transformation scenario beginning with a snow-laden forest inhabited by polar bears, penguins, and deer. The landscape progressively transitions from a winter ecosystem to a lush, verdant woodland, demonstrating ecological succession.
- River: A tranquil riverine setting positioning participants within a minimally intrusive watercraft. The environment accentuates subtle ecological interactions, featuring delicate lotus flowers, riparian vegetation, and microscopic aquatic fauna, creating a contemplative natural experience.

### 2.5. Data Collection

After completing the four-week VR intervention, participants took part in face-to-face semi-structured interviews to explore their experiences. The interview methodology employed semi-structured questions designed to elicit in-depth narratives about participants’ engagement with the VR-based stress reduction intervention.

The interviews were conducted by the lead author and two trained research assistants. The research assistants received training in qualitative interviewing techniques from the lead author, with an emphasis on phenomenological inquiry approaches. All interviewers completed practice sessions that were evaluated for consistency before data collection began. Before each interview, participants were provided with detailed information about the study’s purpose and procedures, given opportunities to review consent documentation, and encouraged to ask clarifying questions. Interviews proceeded only after participants had provided informed written consent. All interviews followed the same protocol to ensure a standardized approach. The interview guide was developed collaboratively by the research team and piloted with three individuals not included in the final sample to refine the questions and interviewing approach. The interview guide was structured to examine multiple dimensions of participants’ experiences, including subjective experiences during VR immersion, perceived impacts on stress levels and well-being, emotional and psychological responses to virtual scenarios, mechanisms of stress reduction and relaxation, environmental elements contributing to participant engagement, potential carryover effects and post-intervention mood states, intervention feasibility and acceptability, and comparative perspectives with alternative stress reduction techniques. Supplementary Materials File S2 presents the complete set of questions. Sample questions included the following:

- Please describe your experience during the VR-based stress reduction intervention.
- What thoughts, feelings, or sensations did you experience while immersed in the virtual environment?
- Did you feel a sense of relaxation or calmness during the VR intervention? If so, can you describe what contributed to this feeling?
- Did you notice any carryover effects or changes in your mood, mindset, or stress levels after completing the VR intervention?
- Were there any challenges or limitations you encountered during the VR experience that affected your ability to relax or engage with the intervention?

The interviews were conducted between March and May 2024, lasting between 30 and 45 min each. Participants provided informed consent, confirming their voluntary participation and understanding of the study’s goals and procedures. This approach facilitated an in-depth exploration of participants’ unique experiences within the virtual nature environments, prioritizing rich, qualitative insights. Data confidentiality was maintained through the anonymous coding of all participant information, secure storage of data on encrypted drives, and removal of identifying information from transcripts. Participants were assigned code numbers in all research outputs to protect their identities.

### 2.6. Data Analysis

The study employed thematic analysis following Braun and Clarke’s [47] established six-phase methodological framework using NVivo 14 software. This approach was selected for its methodological flexibility and robust capacity to identify meaningful patterns across qualitative datasets while maintaining theoretical versatility. The software was utilized throughout the analysis process to organize and manage the qualitative data. Specifically, we used the software to create a structured coding system, systematically assign codes to relevant text segments, and organize these codes hierarchically. The software’s data management capabilities allowed us to efficiently retrieve, compare, and reorganize coded content during theme development. The analytical process commenced with comprehensive data familiarization, involving repeated and systematic reading of interview transcripts. Initial coding was conducted inductively, allowing codes to emerge organically from the data rather than constraining them within predetermined analytical frameworks. We generated codes systematically across the entire dataset, capturing salient and interesting features of participants’ narratives. Subsequent phases involved searching for potential themes by collating related codes and gathering relevant data segments. Three researchers critically reviewed these emerging themes about both coded extracts and the comprehensive dataset, ensuring they represented coherent and meaningful patterns that authentically reflected participants’ experiences. Themes were iteratively defined, named, and refined through ongoing analytical engagement.

Code or thematic saturation was a critical consideration in sample size determination [48]. Thematic saturation refers to the stage in the data analysis process where repetitive codes or themes are identified and no substantially new information or relationships between them emerge [46]. The sample size of participants was strategically selected to ensure comprehensive data exploration while achieving thematic saturation. As new participants’ data began to replicate existing themes with minimal novel insights, it signaled the point of saturation, indicating that the sample size was sufficient to capture the breadth and depth of participants’ experiences.

## 3. Results

**Figure 1:**
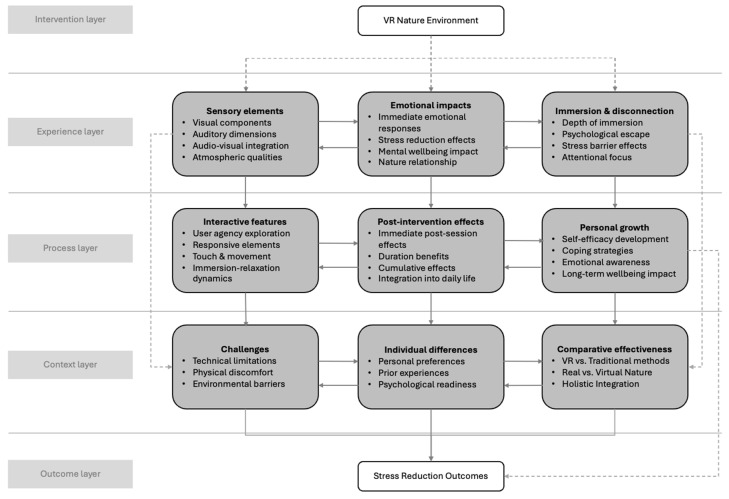
Conceptual model of a virtual nature environment for stress reduction effects among young adults. Solid arrows indicate direct influences; dotted arrows indicate cross-layer influences.

The study included 35 young adults between 19 and 29 years of age (M = 22.2, SD = 1.93) recruited from universities. In total, 24 were female, and 11 were male. Most participants (n = 26) balanced full-time university studies with part-time employment, while seven were exclusively engaged in full-time university studies, and two maintained full-time employment. The overview information of the participants is shown in Table S1. All 35 participants completed all four weekly VR sessions as scheduled. Analysis of the VR nature experience data identified a layered conceptual framework comprising five distinct levels: intervention, experience, process, context, and outcome layers. Each layer encompasses specific thematic elements that collectively explain how young adults interact with and respond to virtual natural environments for relaxation purposes. These elements exhibit bidirectional relationships both within and across layers, suggesting a complex, dynamic process rather than a linear progression through the VR experience. Figure 1 illustrates the conceptual model representing these findings.

The first layer, known as the intervention layer, consists of the foundational VR nature experience, which includes the technical aspects of the intervention itself. Following this is the experience layer, which encompasses the immediate perceptual and affective elements that are directly elicited during the engagement with the VR environment. The process layer represents the ongoing mechanisms through which the experience develops and evolves. The context layer involves the broader situational elements that either moderate or mediate the experience and its effects. Finally, the outcome layer captures the resultant stress reduction effects that emerge from the complete experiential process.

The integrated analysis of these layers reveals how attention restoration mechanisms and stress recovery processes operate in tandem through the VR nature experience to facilitate relaxation outcomes. The identified layered model demonstrates clear alignments with both ART and SRT, with different layers activating specific theoretical mechanisms.

### 3.1. Intervention Layer

The intervention layer consists of the VR nature experience itself. This served as the foundational element from which all other thematic components emerged. Participants universally recognized this core element as the catalyst for subsequent experiences, though their specific responses to this intervention varied considerably. As one participant noted:

*“The VR sessions were structured the same way each time, but my reaction to them changed across the four sessions as I became more comfortable with the technology and more receptive to the environments.”*

(P11, Female, Age 21)

The intervention directly influenced all components within the experience layer, initiating a cascade of perceptual, emotional, and cognitive responses.

### 3.2. Experience Layer

The experience layer encompasses the immediate perceptual and affective elements directly elicited during engagement with the VR environment. Three primary themes were identified at this layer: sensory elements, emotional impact, and immersion and disconnection. Elements within the experience layer primarily activated the theoretical mechanisms posited by both ART and SRT.

#### 3.2.1. Sensory Elements

Sensory elements emerged as a significant component of participants’ immediate experience, comprising visual components, auditory dimensions, audio–visual integration, interactive features, atmospheric qualities, and sensory limitations. The sensory elements theme facilitated ART’s “fascination” component through engaging visual and auditory features that captured attention effortlessly. Concurrently, these same elements triggered SRT’s evolutionary-based psychophysiological responses to natural stimuli.

Participants consistently emphasized the primacy of visual and auditory sensations in creating a convincing natural environment:

*“The visual content easily brought me into a peaceful and relaxed atmosphere…The music was peaceful and played at a calm pace.”*

(P5, Male, Age 22)

The synchronization between visual and auditory elements substantially influenced the perceived authenticity of the experience, with well-integrated sensory components enhancing relaxation whilst misaligned elements disrupted immersion:

*“The harmonious combination of visual and auditory stimuli also plays a key role in promoting relaxation. Soft, serene melodies intertwined with soothing natural sounds calmed my mind and freed me from stress.”*

(P30, Female, Age 20)

Participants sometimes highlighted sensory limitations, particularly the absence of tactile and olfactory components, as factors that diminished their immersion:

*“Multi-sensory participation makes the whole thing seem more real, so that attention can be more focused.”*

(P24, Female, Age 22)

#### 3.2.2. Emotional Impact

Participants reported a broad spectrum of emotional responses to the VR nature environments, encompassing immediate emotional reactions, stress reduction effects, impacts on overall mental wellbeing, personal growth dimensions, and emotional relationships with nature. Immediate responses typically involved feelings of calm, wonder, or occasionally unease:

*“I remember seeing many different kinds of fish swimming in front of me, and there was a remnant of a pirate ship nearby. It was all so fascinating because I had never seen an underwater landscape like that before, although when I was diving in a real undersea.”*

(P20, Female, Age 20)

Emotional connections to nature were frequently reported, often linked to nostalgic associations with previous nature experiences:

*“I could listen to ambient sounds, such as other birds singing, winds blowing, trees rustling. It recalled my memory of vacation in Bangkok, and I was on the beach with lovely sunset.”*

(P1, Male, Age 24)

These emotional responses exhibited bidirectional relationships with sensory elements, as emotional states influenced the perception of environmental features, whilst sensory components triggered specific affective states.

#### 3.2.3. Immersion and Disconnection

The depth of immersion in VR environments and psychological disconnection from real-world concerns constituted a third major theme within the experience layer. This encompasses depth of immersion, psychological escape, stress barrier effects, attentional focus dynamics, and immersion-relaxation relationships. Immersion and disconnection components strongly aligned with ART’s “being away” and “extent” dimensions, creating psychological distance from stressors and providing sufficiently rich environments to occupy attention.

Participants described varying levels of presence within the virtual environments:

*“I am fully immersed in the virtual world, where I can relax and start to enjoy the music and scenery. The music and scenery in the virtual world create a feeling of being there.”*

(P6, Female, Age 25)

The psychological escape afforded by immersion functioned as a barrier against intrusive stressful thoughts:

*“When I put on the VR headset and enter the virtual world, I can temporarily forget about the stresses, worries, and fatigue of real-life. In the virtual environment, I can freely explore, adventure, and engage in various activities, which brings me a feeling of relaxation and detachment.”*

(P7, Female, Age 27)

The relationship between immersion depth and relaxation outcomes was not strictly linear; participants noted a threshold effect whereby minimal immersion yielded minimal benefits, but beyond a certain threshold, deeper immersion did not necessarily produce proportionally greater relaxation.

### 3.3. Process Layer

The process layer represents the ongoing mechanisms through which the experience develops and evolves across and between sessions. Three primary themes emerged at this level: interactive features, post-intervention effects, and personal growth. The process layer features demonstrated how these theoretical mechanisms develop over time, with personal growth reflecting the sustained benefits predicted by both theories when natural environments are experienced repeatedly.

#### 3.3.1. Interactive Features

Participants’ ability to interact with the virtual environment emerged as a key process element, encompassing user agency in exploration, responsive environmental elements, touch and movement capabilities, and immersion-relaxation dynamics. The capacity to navigate freely through environments enhanced feelings of agency and engagement:

*“Additionally, the interactive elements in the scenarios were also very helpful for stress reduction. As I explored each scenario, I felt completely focused on the present moment.”*

(P15, Female, Age 24)

Interactive features exhibited bidirectional relationships with aspects of the experience layer, particularly immersion and sensory elements, with greater interactivity typically enhancing immersion.

#### 3.3.2. Post-Intervention Effects

Participants reported various temporal effects following VR sessions, including immediate post-session effects, duration of benefits, cumulative effects of multiple sessions, integration into daily life, long-term wellbeing impacts, and dose–response relationships. Immediate effects typically involved continued feelings of relaxation:

*“The VR intervention had a remarkably durable impact, with the calming and restoring effects unforgettable even after the immersive experiences.”*

(P17, Female, Age 24)

The duration of relaxation effects varied considerably among participants, from minutes to days:

*“Although I experienced stress reduction during the VR intervention, I did not observe any carryover effects on my mood, mindset, or stress levels… stress reduction often requires long-term interventions in order to have sustained effects”*

(P2, Male, Age 22)

Participants frequently reported cumulative benefits across multiple sessions, suggesting a learning or conditioning effect:

*“However, this only lasted for a while, I soon went back to thinking about real-life worries. But every time I think back to the VR experience and the pictures, I feel relaxed.”*

(P18, Female, Age 21)

#### 3.3.3. Personal Growth

Some participants identified elements of personal development resulting from repeated engagement with VR nature, including self-efficacy development, coping strategies, emotional awareness, and long-term wellbeing impacts. Several reported transferring relaxation techniques learned in VR to non-VR contexts:

*“This successful experience made me feel more confident and enhanced my self-efficacy as I understood I could achieve my goals even if they were challenging. Through recalling this scenario, my stress level was lowered as I became more confident in overcoming my anxiety about deadlines.”*

(P13, Female, Age 22)

Increased awareness of emotional states was noted by several participants:

*“This mindset of being fully present and aware carried, helping me to have more clarity and focus.”*

(P15, Female, Age 24)

### 3.4. Context Layer

The context layer encompasses broader situational elements that moderate or mediate the VR nature experience and its effects. Three primary themes were identified: challenges, individual differences, and comparative effectiveness.

#### 3.4.1. Challenges

Participants reported various obstacles affecting their VR nature experience, including technical limitations, physical discomfort, environmental barriers, and psychological engagement issues. Technical constraints such as resolution limitations or tracking problems frequently disrupted immersion:

*“At the same time, the scene is not clear enough, the limitation of the resolution will decrease the quality of VR experience.”*

(P4, Male, Age 22)

Physical discomfort, particularly from extended headset wear, emerged as a significant barrier:

*“Prolonged use led to discomfort and pain in my head and neck.”*

(P2, Male, Age 22)

Environmental distractions in the physical space where VR was experienced also diminished effectiveness:

*“Hearing other talking in the next room made it hard to stay immersed in the beach environment.”*

(P9, Female, Age 22)

#### 3.4.2. Individual Differences

Substantial variation in preferences and responses was observed, encompassing personal preferences, prior experiences, and psychological readiness. Preferences for specific environments varied considerably:

*“Personally, I prefer virtual scenarios of snow-capped mountains and forests because they are interesting and novel to me.”*

(P23, Female, 23)

Previous experience with both nature and technology influenced participants’ responses:

*“While VR can simulate nature’s visual and auditory effects, it cannot replicate the smells of the outdoors.”*

(P13, Female, 22)

Individual psychological factors mediated effectiveness:

*“I encountered getting motion nausea from prolonged immersion in virtual reality.”*

(P25, Male, 23)

#### 3.4.3. Comparative Effectiveness

Participants frequently evaluated VR nature against alternative relaxation approaches, encompassing VR versus traditional methods, real versus virtual nature experiences, and integration with holistic approaches. Direct comparisons with other relaxation techniques yielded varied perspectives:

*“…the effectiveness of the virtual reality-based intervention is higher than that of mindfulness since I did not feel relaxed or notice any changes after experiencing mindfulness.”*

(P12, Female, 19)

Most participants viewed VR nature as complementary to, rather than a replacement for, real nature exposure:

*“I consciously choose to balance my stress reduction techniques between outdoor physical activities and immersive VR experiences.”*

(P34, Male, Age 21)

### 3.5. Outcome Layer

The outcome layer represents the resultant stress reduction effects emerging from the complete experiential process. This culminating layer manifested primarily through psychological and physiological relaxation responses:

*“After the VR sessions, I temporary got into relaxation, loosen up and keeping gently mood for half day. Although the due assignment still exists, the lasting effect of happiness altered my attitude.”*

(P1, Male, Age 24)

Participants consistently reported reductions in perceived stress following VR nature sessions, though the magnitude and duration of these effects varied considerably based on factors from preceding layers:

*“My mood improved after a month of immersing myself in virtual reality nature.”*

(P7, Female, Age 27)

This layered conceptual model illustrates how VR nature experiences facilitate stress reduction through the parallel activation of attention restoration and psychophysiological recovery pathways, moderated by individual and contextual factors.

## 4. Discussion

The thematic analysis yields substantial insights into the phenomenological dimensions of young adults’ engagement with virtual natural environments for stress reduction purposes. Through rigorous thematic analysis, we have constructed a multidimensional conceptual framework comprising interconnected intervention, experience, process, context, and outcome layers. This sophisticated framework illuminates the complex psychophysiological mechanisms through which VR nature experiences attenuate stress responses while simultaneously elucidating the intricate interactions among sensory, emotional, and cognitive dimensions. These findings facilitate a more nuanced understanding of both the immediate experiential phenomena and the broader contextual determinants influencing the therapeutic efficacy of virtual nature environments for psychological wellbeing.

### 4.1. Comparison with the Existing Literature

Our findings both corroborate and substantially extend previous empirical work on VR nature for psychological wellbeing. Consistent with Browning et al. [43], we found that virtual natural environments can induce measurable reductions in perceived stress. However, while previous investigations have primarily demonstrated these effects using quantitative physiological measurements or psychometric self-report instruments [16,17], our qualitative approach offers more nuanced insights into the subjective experiential dimensions underlying these effects and the multifaceted factors moderating them.

The critical importance of sensory integration aligns with findings of da Silveira et al. [49], who identified audiovisual synchronicity as essential for establishing presence in virtual environments. Our investigation extends this understanding by delineating specific sensory elements that most effectively promote psychophysiological relaxation and by elucidating the bidirectional relationship between sensory perception and emotional state. The synchronization among multisensory components substantively influenced both the perceived authenticity of the experience and its resultant therapeutic efficacy.

Regarding immersion, our thematic analysis revealed participants’ experiences of psychological engagement with virtual environments. Participants described their experiences of being fully present in the virtual world, emphasizing a sense of relaxation and enjoyment of the surrounding music and scenery [50]. Many participants articulated how the virtual environment provided a temporary escape from real-life stresses, worries, and fatigue, aligning with previous research on psychological disengagement in immersive technologies [51]. These narratives highlight the subjective nature of immersion, illuminating how participants conceptualized and experienced their engagement with virtual nature environments [52]. While these insights provide rich, contextualized understanding, further research would be needed to explore the complexities of immersive experiences in virtual settings. Technical constraints emerged as significant barriers to effective stress reduction, aligning with challenges noted in previous research [53]. Participants frequently mentioned that resolution limitations and tracking issues disrupted their immersion. Physical discomfort was also identified as a notable barrier. These observations highlight the importance of addressing technological and physiological constraints when implementing VR nature interventions.

### 4.2. Interconnected Nature of Thematic Elements

The thematic analysis revealed complex interconnections among the identified themes, highlighting the multidimensional nature of VR experiences. Rather than operating as isolated phenomena, the themes exhibited intricate bidirectional relationships that substantially influenced participants’ overall experiences and therapeutic outcomes. This resonates with Maya-Jariego et al. [54], who emphasize that individual psychological elements rarely function in isolation but rather operate within sophisticated relational networks.

Within the experience layer, particularly robust interconnections were observed between sensory elements and emotional responses. When participants encountered well-integrated audiovisual stimuli, these sensory components triggered positive emotional states, which subsequently enhanced their perceptual sensitivity to environmental details, establishing what several participants described as an immersive “flow state”. As most participants stated, the visual content created a peaceful and relaxed atmosphere, complemented by music that was soothing and played at a calm pace. This finding supports Kurth et al. [55], who identified similar reciprocal relationships between sensory processing and emotional states in experiential contexts.

The relationship between emotional impact and immersion depth emerged as particularly salient. Participants who reported strong positive emotional responses typically described deeper immersive experiences, while those who struggled with immersion frequently reported diminished emotional engagement. This observation resonates with Hess et al.’s [56] findings regarding the bidirectional relationship between emotional states and situational engagement. The analysis also revealed insights regarding the threshold effect of immersion on therapeutic outcomes. While we did not employ standardized immersion measures, participants’ narratives indicated an observable immersion threshold necessary for therapeutic benefit. This threshold appeared to be reached when participants felt psychologically transported to the virtual environment. The relationship between immersion depth and relaxation outcomes followed a non-linear pattern—minimal immersion yielded minimal benefits, but beyond a certain threshold, increasingly deeper immersion did not necessarily produce proportionally greater relaxation. Technical limitations proved tolerable to a certain extent; minor resolution issues did not significantly impair therapeutic effects, while persistent tracking problems consistently disrupted the immersive experience and diminished benefits. Importantly, our findings suggest that even partially immersive experiences appeared sufficient to produce stress reduction effects, indicating that perfect immersion is not necessary to achieve therapeutic outcomes. This observation has significant practical implications, suggesting that moderate-quality VR systems may be adequate for therapeutic applications, potentially increasing the accessibility of such interventions.

The analysis further revealed pronounced cross-layer connections between process elements and experiential components. Interactive features directly influenced both emotional impact and immersion quality, while simultaneously being constrained by technical limitations. For instance, most participants noted that the interactive elements in the scenarios were helpful for stress reduction, enabling them to feel completely focused on the present moment as they explored each scenario. However, technical constraints often undermined this potential benefit, with resolution limitations and tracking problems disrupting the immersive experience. Individual differences exhibited notable moderating effects on the relationship between emotional impact and post-intervention outcomes. Participants’ environmental preferences, prior experiences with nature, and technological familiarity significantly influenced both immediate responses and the duration of therapeutic benefits. This finding supports de Villiers et al.’s [57] assertion that individual differences substantially moderate experiential outcomes in therapeutic contexts.

These interrelationships offer important explanatory power regarding the considerable variability in outcomes observed across participants. When sensory elements, emotional responses, immersive qualities, and interactive features functioned synergistically, participants reported stress reduction benefits. Conversely, deficiencies in one area—such as technical limitations disrupting immersion—often cascaded through the experiential system, substantially diminishing therapeutic effectiveness. This systemic perspective aligns with Smith and Conrey [58], who emphasized the importance of considering interactive effects rather than isolated variables when examining complex experiential phenomena.

### 4.3. Theoretical Implications

The thematic analysis yielded findings that align remarkably with established theoretical frameworks while simultaneously extending their application to virtual nature contexts. Both ART and SRT received substantial empirical validation through participants’ reported experiences, with our findings supporting key mechanisms from both theories while identifying important VR-specific considerations.

The “immersion and disconnection” theme demonstrated clear correspondence with ART’s “being away” component, with participants describing psychological detachment from everyday concerns that Kaplan [31] identified as essential for attentional resource recovery. This psychological separation created what we conceptualized as a “stress barrier”, temporarily shielding individuals from intrusive thoughts. Regarding ART’s “fascination” dimension, participants reported that specific environmental features captured their attention effortlessly, exemplifying the “soft fascination” central to restoration. For the “extent” dimension, participants’ descriptions of immersive depth supported its relevance in virtual contexts, though sensory limitations in current VR technology diminished their environmental extent perception.

SRT’s theoretical premises also received substantial support through the “emotional impact” theme. Participants reported immediate affective responses to virtual environments, aligning with Ulrich’s [38] proposition that such reactions represent evolutionarily prepared mechanisms. The specific natural features that elicited positive emotional responses—water elements, expansive vistas, and sheltered spaces—directly correspond with those SRT identifies as historically signaling environmental safety and resource availability. Their reports of physiological responses further supported SRT’s unwinding mechanisms, though technological mediation moderated these effects. Notably, multisensory integration significantly influenced both theoretical processes: well-synchronized audiovisual elements enhanced attentional capture and emotional response, while sensory inconsistencies disrupted restoration.

Beyond supporting these established frameworks, our analysis suggested important theoretical extensions. Rather than operating independently, ART and SRT mechanisms functioned synergistically in virtual contexts, with immersion intensifying emotional responses and positive affective states enhancing attentional fascination. The “process layer” findings revealed temporal dynamics largely unaddressed in current theoretical formulations, suggesting that restoration may constitute a developable skill that improves with practice, while the “personal growth” theme indicated that VR nature experiences may facilitate broader psychological development, including enhanced emotional awareness and expanded coping repertoires.

These findings contribute meaningfully to theoretical understanding by demonstrating that while ART and SRT maintain explanatory power in virtual contexts, their application requires consideration of technology-mediated natural environments’ unique affordances and limitations, particularly regarding the integration of attentional and emotional processes and the developmental aspects of restoration across multiple experiences.

### 4.4. Practical Implications

The study unveils multifaceted implications for implementing VR nature experiences across therapeutic, workplace, and educational domains. Critical findings emphasize sensory integration as a pivotal factor in effectiveness, with synchronized audiovisual elements enhancing immersion. Notably, a threshold effect was observed in immersion depth and therapeutic outcomes, suggesting that moderately immersive applications can be therapeutically effective without requiring maximal technological sophistication. Individual differences emerged as a crucial consideration, highlighting the necessity for personalized VR experiences. Pronounced variations in environmental preferences and psychophysiological responses underscore the limitations of standardized approaches. Physical comfort also proved paramount, with headset-related discomfort potentially compromising the therapeutic experience. In mental health contexts, VR nature demonstrates potential as an adjunctive therapeutic modality, particularly for conditions characterized by rumination. The identified “stress barrier” effect—temporarily inhibiting intrusive cognitions—offers promising intervention mechanisms. Workplace applications suggest efficient stress reduction during work breaks, with potential transferable relaxation techniques. Educational settings may benefit from dual cognitive and emotional regulation applications. VR nature experiences show potential for stress reduction and potential cognitive performance enhancement through attention restoration mechanisms. The identified personal growth outcomes, including improved emotional awareness and expanded coping strategies, suggest broader applications in social–emotional learning. These practical implications collectively provide a nuanced framework for VR nature experience implementation, emphasizing the critical importance of sensory integration, individual customization, physical comfort, and progressive design elements to optimize therapeutic effectiveness across diverse contexts.

### 4.5. Limitations

While this investigation provides valuable insights into young adults’ experiences with VR nature for stress reduction, several methodological limitations warrant acknowledgement. Our sample of 35 young adults (aged 19–29) from primarily university settings limits generalizability to other age groups whose relationships with nature and technology might differ significantly. We did not systematically assess participants’ baseline nature connectedness, previous nature exposure patterns, residential settings (urban versus rural), or personal preferences for nature exploration. These factors likely influenced how participants experienced and responded to the virtual environments. These individual differences in nature relationships represent important potential moderators that were not controlled for in the current study. An additional methodological consideration is the potential influence of the ‘novelty effect’ on our findings. Most participants reported limited or no prior experience with VR technology before this study, which may have heightened their initial interest and engagement with the intervention. This novelty factor could have influenced participants’ evaluations of the experience, potentially leading to an overestimation of the intervention’s effectiveness during the four weeks. The observed cumulative benefits across sessions suggest that the effects extended beyond initial novelty; however, we cannot fully distinguish between therapeutic benefits and the psychological impact of engaging with novel technology. 

Additionally, varying familiarity with VR technology likely influenced participants’ immersion and emotional responses, suggesting future studies should control for prior VR exposure. Beyond demographic constraints, technical limitations of consumer-grade VR technology impacted the experience. The absence of olfactory and tactile stimuli reduced perceived authenticity, while resolution limitations and tracking inconsistencies disrupted immersion for some participants. As VR technology evolves, incorporating multisensory elements may reveal more pronounced therapeutic effects. A significant methodological limitation is the absence of a control condition featuring non-nature VR content. Without comparing nature-based environments to alternative virtual content (such as urban scenes or abstract environments), we cannot definitively determine whether the observed stress reduction effects stem specifically from nature exposure or from the general experience of VR immersion. While our findings align with Stress Recovery Theory’s predictions regarding nature’s restorative properties, the experimental design does not allow us to isolate nature as the causal factor.

From a methodological perspective, our qualitative approach provides rich experiential data but cannot determine effect magnitude across larger populations. The brief four-session structure may not capture longer-term adaptation patterns. Furthermore, we relied on self-reported experiences without objectively verifying physiological changes, which expectancy effects may have influenced. Future research would benefit from integrating physiological measurements with qualitative reports to provide more comprehensive evidence of virtual nature’s impact.

### 4.6. Suggestions for Future Research

Several promising directions for future research emerge from our findings and methodological limitations. First, the identified cumulative effects and personal growth outcomes suggest substantial value in longitudinal investigations. Extended studies examining VR nature use over months rather than weeks could determine whether benefits plateau, continue to increase, or diminish with extended use. Such research might also identify possible developmental stages or patterns in how individuals incorporate VR nature into their wellness practices over time. Future research should control for prior VR exposure and conduct longer-term interventions to determine whether reported benefits persist beyond the novelty period. Studies comparing VR-naive participants with those who have substantial prior VR experience would be particularly valuable in isolating the specific therapeutic mechanisms from technology-related excitement.

Moreover, our findings regarding individual variations in preferences and responses highlight the need for more targeted research into moderating factors. Studies specifically examining how personality traits, nature connectedness, previous nature exposure, residential environment (urban versus rural), cultural background, or frequency of actual nature interaction influence VR nature’s effectiveness could inform more personalized implementation strategies. Developing standardized assessments of baseline nature relationships would allow researchers to stratify participants and potentially identify which individuals might benefit most from VR nature interventions versus traditional nature exposure.

Additionally, future research would benefit from combining qualitative phenomenological explorations with quantitative physiological and cognitive measures. Such mixed-methods approaches could verify subjective reports of relaxation with objective indicators (e.g., cortisol levels, heart rate variability, electrodermal activity) while maintaining rich experiential data. This methodological integration would strengthen the evidence base for VR nature interventions and potentially identify physiological markers of differential response patterns. Future research should implement comparative designs with multiple VR content types to differentiate between nature-specific effects and general VR-induced relaxation responses.

Furthermore, while our participants frequently compared VR nature to other relaxation modalities, systematic comparative studies directly contrasting VR nature with established approaches (e.g., mindfulness meditation, exposure to photographic stimuli, physical nature encounters) would clarify its relative advantages and limitations. Such research should examine not only which approaches demonstrate greater efficacy but also for whom and under what circumstances each approach might be optimally indicated. Finally, experimental studies systematically manipulating specific design elements (e.g., levels of interactivity, sensory features, environmental typologies) could build upon our conceptual model to determine which aspects most significantly influence therapeutic outcomes. Such research could establish evidence-based design guidelines specifically for therapeutic VR nature applications, potentially diverging from general VR design principles.

Future investigations should explore hardware modifications to enhance user comfort during extended therapeutic VR sessions. Potential solutions include supplementary support systems like Elite Straps that better distribute headset weight and reduce pressure points, cushioned facial interfaces to improve comfort, and counterweights to balance the front-heavy nature of most headsets. Software enhancements such as minimizing or deactivating heads-up display (HUD) elements that can intrude on nature immersion would be valuable, as would improve tracking algorithms to reduce disorientation. Additionally, research into optimizing session duration to balance therapeutic efficacy against physical comfort could establish evidence-based guidelines for clinical implementation. These practical considerations are critical as VR nature interventions move from experimental to applied contexts, particularly for populations who might benefit from extended or repeated sessions.

## 5. Conclusions

This investigation has illuminated the complex phenomenology of how young adults experience and respond to virtual natural environments for stress reduction, revealing a multifaceted, layered process involving sensory, emotional, cognitive, and contextual elements. Our findings demonstrate that VR nature experiences can facilitate meaningful stress reduction through mechanisms aligned with both ART and SRT, though with important modifications specific to the virtual context. VR nature experiences represent a promising approach for promoting wellbeing among young adults, offering an accessible means of obtaining many of nature’s psychological benefits even when physical nature access is constrained. By understanding the mechanisms through which these experiences operate—as illuminated by our layered conceptual model—researchers, designers, and practitioners can develop more effective applications across mental health, workplace wellness, and educational contexts, potentially contributing to a broader therapeutic toolkit for addressing stress-related challenges in contemporary society.

## Data Availability

The data supporting the conclusions of this article will be made available by the authors on request.

## Supplementary Materials

The following supporting information can be downloaded at: https://www.mdpi.com/article/10.3390/healthcare13080895/s1, File S1: Virtual natural environments; File S2: Questions for interview; Table S1: General information of interviewees (n = 35).

## Abbreviations

The following abbreviations are used in this manuscript:

VR	Virtual Reality
ART	Attention Restoration Theory
SRT	Stress Recovery Theory
WHO	World Health Organization

## References

[1] Shek D.T.L., Dou D., Zhu X. (2022). Prevalence and correlates of mental health of university students in Hong Kong: What happened one year after the occurrence of COVID-19?. Front. Public Health.

[2] Ng P.Y., Yang S., Chiu R. (2024). Features of emerging adulthood, perceived stress and life satisfaction in Hong Kong emerging adults. Curr. Psychol..

[3] Wong S.M.Y., Lam B.Y.H., Wong C.S.M., Lee H.P.Y., Wong G.H.Y., Lui S.S.Y., Chan K.T., Wong M.T.H., Chan S.K.W., Chang W.C. (2021). Measuring subjective stress among young people in Hong Kong: Validation and predictive utility of the single-item subjective level of stress (SLS-1) in epidemiological and longitudinal community samples. Epidemiol. Psychiatr. Sci..

[4] World Health Organization Stress. https://www.who.int/news-room/questions-and-answers/item/stress.

[5] Klainin-Yobas P., Vongsirimas N., Ramirez D.Q., Sarmiento J., Fernandez Z. (2021). Evaluating the relationships among stress, resilience and psychological well-being among young adults: A structural equation modelling approach. BMC Nurs..

[6] Shek D.T.L., Chai W.Y., Wong T., Zhou K. (2023). Stress and depressive symptoms in university students in Hong Kong under the pandemic: Moderating effect of positive psychological attributes. Front. Psychol..

[7] Fruehwirth J.C., Weng A.X., Perreira K.M. (2024). The effect of social media use on mental health of college students during the pandemic. Health Econ..

[8] Steare T., Gutiérrez Muñoz C., Sullivan A., Lewis G. (2023). The association between academic pressure and adolescent mental health problems: A systematic review. J. Affect. Disord..

[9] Zisopoulou T., Varvogli L. (2023). Stress management methods in children and adolescents: Past, present, and future. Horm. Res. Paediatr..

[10] Braun P., Atik E., Guthardt L., Apolinário-Hagen J., Schückes M. (2023). Barriers to and facilitators of a blended cognitive behavioral therapy program for depression and anxiety based on experiences of university students: Qualitative interview study. JMIR Form. Res..

[11] Gagné T., Nandi A., Schoon I. (2021). Time trend analysis of social inequalities in psychological distress among young adults before and during the pandemic: Evidence from the UK Household Longitudinal Study COVID-19 waves. J. Epidemiol. Community Health.

[12] Wilkinson P.O., Qiu T., Jesmont C., Neufeld S.A.S., Kaur S.P., Jones P.B., Goodyer I.M. (2022). Age and gender effects on non-suicidal self-injury, and their interplay with psychological distress. J. Affect. Disord..

[13] Payne E.A., Loi N.M., Thorsteinsson E.B. (2020). The restorative effect of the natural environment on university students’ psychological health. J. Environ. Public Health.

[14] Jarvis I., Gergel S., Koehoorn M., van den Bosch M. (2020). Greenspace access does not correspond to nature exposure: Measures of urban natural space with implications for health research. Landsc. Urban Plan..

[15] Bell I. (2020). Virtual reality as a clinical tool in mental health research and practice. Dialogues Clin. Neurosci..

[16] Björling E.A., Sonney J., Rodriguez S., Carr N., Zade H., Moon S.H. (2022). Exploring the effect of a nature-based virtual reality environment on stress in adolescents. Front. Virtual Real..

[17] Chan S.H.M., Qiu L., Esposito G., Mai K.P., Tam K.-P., Cui J. (2021). Nature in virtual reality improves mood and reduces stress: Evidence from young adults and senior citizens. Virtual Real..

[18] Maples-Keller J.L., Bunnell B.E., Kim S.-J., Rothbaum B.O. (2017). The use of virtual reality technology in the treatment of anxiety and other psychiatric disorders. Harv. Rev. Psychiatry.

[19] Javaid S.F., Hashim I.J., Hashim M.J., Ahmed A., Marashi M., Bertolino L. (2023). Epidemiology of anxiety disorders: Global burden and sociodemographic associations. Middle East Curr. Psychiatry.

[20] Barker M.M., Beresford B., Bland M., Fraser L.K. (2019). Prevalence and incidence of anxiety and depression among children, adolescents, and young adults with life-limiting conditions: A systematic review and meta-analysis. JAMA Pediatr..

[21] Li K., Cooke E.M., Zheng Y. (2024). Dynamic links between daily anxiety symptoms and young adults’ daily well-being. Anxiety Stress Coping.

[22] Lipson S.K., Zhou S., Abelson S., Heinze J., Jirsa M., Morigney J., Patterson A., Singh M., Eisenberg D. (2022). Trends in college student mental health and help-seeking by race/ethnicity: Findings from the national healthy minds study, 2013–2021. J. Affect. Disord..

[23] Lane J.A., Leibert T.W., Goka-Dubose E. (2017). The impact of life transition on emerging adult attachment, social support, and well-being: A multiple-group comparison. J. Couns. Dev..

[24] Teixeira S., Ferré-Grau C., Canut T.L., Pires R., Carvalho J.C., Ribeiro I., Sequeira C., Rodrigues T., Sampaio F., Costa T. (2022). Positive mental health in university students and its relations with psychological vulnerability, mental health literacy, and sociodemographic characteristics: A descriptive correlational study. Int. J. Environ. Res. Public Health.

[25] Ahad A.A., Sanchez-Gonzalez M., Junquera P. (2023). Understanding and addressing mental health stigma across cultures for improving psychiatric care: A narrative review. Cureus.

[26] Mazgelytė E., Rekienė V., Dereškevičiūtė E., Petrėnas T., Songailienė J., Utkus A., Chomentauskas G., Karčiauskaitė D. (2021). Effects of virtual reality-based relaxation techniques on psychological, physiological, and biochemical stress indicators. Healthcare.

[27] Velana M., Sobieraj S., Digutsch J., Rinkenauer G. (2022). The advances of immersive virtual reality interventions for the enhancement of stress management and relaxation among healthy adults: A systematic review. Appl. Sci..

[28] Chen W.-W., Xu G., Wang Z., Mak M.C.K. (2020). Unhappy us, unhappy me, unhappy life: The role of self-esteem in the relation between adult attachment styles and mental health. Curr. Psychol..

[29] Qin N., Li Y., Duan Y.L., Luo Y.T., Li J., Cao H., Zhou X., Wang Y.Q., Yang P.T., Xie J.F. (2024). Associations between healthy lifestyle behavioral patterns and mental health problems: A latent class analysis of 161,744 Chinese young adults. J. Affect. Disord..

[30] Kaplan R., Kaplan S. (1989). The Experience of Nature: A Psychological Perspective.

[31] Kaplan S. (1995). The restorative benefits of nature: Toward an integrative framework. J. Environ. Psychol..

[32] Slavich G.M., Irwin M.R. (2014). From stress to inflammation and major depressive disorder: A social signal transduction theory of depression. Psychol. Bull..

[33] Stevenson M.P., Schilhab T., Bentsen P. (2018). Attention restoration theory II: A systematic review to clarify attention processes affected by exposure to natural environments. J. Toxicol. Environ. Health B Crit. Rev..

[34] Beverly E., Hommema L., Coates K., Duncan G., Gable B., Gutman T., Love M., Love C., Pershing M., Stevens N. (2022). A tranquil virtual reality experience to reduce subjective stress among COVID-19 frontline healthcare workers. PLoS ONE.

[35] Dozio N., Maggioni E., Pittera D., Gallace A., Obrist M. (2021). May I smell your attention: Exploration of smell and sound for visuospatial attention in virtual reality. Front. Psychol..

[36] Finkler W., Vlietstra L., Waters D.L., Zhu L., Gallagher S., Walker R., Forlong R., van Heezik Y. (2025). Virtual nature and well-being: Exploring the potential of 360° VR. Appl. Psychol. Health Well-Being.

[37] Pizzoli S.F.M., Mazzocco K., Triberti S., Monzani D., Alcañiz Raya M.L., Pravettoni G. (2019). User-centered virtual reality for promoting relaxation: An innovative approach. Front. Psychol..

[38] Ulrich R.S., Altman I., Wohlwill J.F. (1983). Aesthetic and affective response to natural environment. Behavior and the Natural Environment.

[39] Ulrich R.S., Simons R.F., Losito B.D., Fiorito E., Miles M.A., Zelson M. (1991). Stress recovery during exposure to natural and urban environments. J. Environ. Psychol..

[40] Scott E.E., LoTemplio S.B., McDonnell A.S., McNay G.D., Greenberg K., McKinney T., Uchino B.N., Strayer D.L. (2021). The autonomic nervous system in its natural environment: Immersion in nature is associated with changes in heart rate and heart rate variability. Psychophysiology.

[41] Joye Y., van den Berg A. (2011). Is love for green in our genes? A critical analysis of evolutionary assumptions in restorative environments research. Urban For. Urban Green..

[42] Chiang Y.-C., Li D., Jane H.-A. (2017). Wild or tended nature? The effects of landscape location and vegetation density on physiological and psychological responses. Landsc. Urban Plan..

[43] Browning M.H.E.M., Mimnaugh K.J., van Riper C.J., Laurent H.K., LaValle S.M. (2020). Can simulated nature support mental health? Comparing short, single-doses of 360-degree nature videos in virtual reality with the outdoors. Front. Psychol..

[44] Maher L., Dertadian G. (2018). Qualitative research. Addiction.

[45] Tenny S., Brannan J.M., Brannan G.D. (2022). Qualitative study. StatPearls.

[46] Lowe A., Norris A.C., Farris A.J., Babbage D.R. (2018). Quantifying thematic saturation in qualitative data analysis. Field Methods.

[47] Braun V., Clarke V. (2006). Using thematic analysis in psychology. Qual. Res. Psychol..

[48] Naeem M., Ozuem W., Howell K., Ranfagni S. (2024). Demystification and actualisation of data saturation in qualitative research through thematic analysis. Int. J. Qual. Methods.

[49] da Silveira A.C., Spyridonis F., Raisamo R., Covaci A., Ghinea G., Santos C.A.S. (2024). On perceived AV synchronization in 360° multimedia. IEEE Multimed..

[50] Puente-Torre P., Delgado-Benito V., Rodríguez-Cano S., García-Delgado M.Á. (2024). Virtual reality as an interactive tool for the implementation of mindfulness in university settings: A systematic review. Multimodal Technol. Interact..

[51] Piumsomboon T., Ong G., Urban C., Ens B., Topliss J., Bai X., Hoermann S. (2022). Ex-Cit XR: Expert-elicitation and validation of extended reality visualisation and interaction techniques for disengaging and transitioning users from immersive virtual environments. Front. Virtual Real..

[52] Yang T., Lai I.K.W., Fan Z.B., Mo Q.M. (2021). The impact of a 360° virtual tour on the reduction of psychological stress caused by COVID-19. Technol. Soc..

[53] Guo J., Weng D., Fang H., Zhang Z., Ping J., Liu Y., Wang Y. Exploring the differences of visual discomfort caused by long-term immersion between virtual environments and physical environments. Proceedings of the IEEE Conference on Virtual Reality and 3D User Interfaces (VR).

[54] Maya-Jariego I., Letina S., González Tinoco E. (2019). Personal networks and psychological attributes: Exploring individual differences in personality and sense of community and their relationship to the structure of personal networks. Netw. Sci..

[55] Kurth F., Zilles K., Fox P.T., Laird A.R., Eickhoff S.B. (2010). A link between the systems: Functional differentiation and integration within the human insula revealed by meta-analysis. Brain Struct. Funct..

[56] Hess U., Dietrich J., Kafetsios K., Elkabetz S., Hareli S. (2019). The bidirectional influence of emotion expressions and context: Emotion expressions, situational information and real-world knowledge combine to inform observers’ judgments of both the emotion expressions and the situation. Cogn. Emot..

[57] de Villiers B., Lionetti F., Pluess M. (2018). Vantage sensitivity: A framework for individual differences in response to psychological intervention. Soc. Psychiatry Psychiatr. Epidemiol..

[58] Smith E.R., Conrey F.R. (2007). Agent-based modeling: A new approach for theory building in social psychology. Pers. Soc. Psychol. Rev..

