# Supplementary material for: Finding Peace in Pixels: Exploring the Therapeutic Mechanisms of Virtual Nature for Young Adults’ Mental Well-Being
Source: Healthcare (Basel). 2025 Apr 14;13(8):895. doi: 10.3390/healthcare13080895 (PMC12027312; doi:10.3390/healthcare13080895)
Supplement: Supplementary file 1 [file healthcare-13-00895-s001.zip › Supplementary Materials File S2. Questions for interview.pdf]

Supplementary Materials File S2. Questions for interview

1.	Please describe your feelings and feelings during your VR nature experience.
1.1.	What thoughts, feelings, or sensations did you experience while you were immersed in a virtual environment?
2.	How do the scenes you experience in VR (under the sea, in the sky, in snowy forests, in rivers) affect your stress or overall health?
2.1.	Are there any specific scenarios that you find more effective at reducing stress?
3.	Do you feel relaxed or calm during your VR nature experience?
3.1.	If so, can you describe what causes this feeling?
3.2.	If not, can you describe what causes this feeling?
4.	How do the immersive nature of VR nature environments affect your ability to escape real-world stress and focus on relaxation?
5.	Are there any elements or features in nature scenes (such as sounds, visuals, or interactive elements) that you find particularly helpful or enjoyable in reducing stress?
6.	After completing the VR nature experience, did you notice any changes in your mood, state of mind, or stress levels?
6.1.	If yes, please elaborate.
7.	During your VR nature experience, did you encounter any limitations that affected your relaxation or participation in the experience?
8.	Do you think VR is acceptable and feasible for stress reduction?
8.1.	Why or why not?
9.	Do you think VR is better for reducing stress than other stress-reducing methods or measures you have tried before?
9.1.	Why? Please explain more.
10.	Do you have any other feedback or suggestions for VR-based stress relief experiences?
