# Supplementary material for: Finding Peace in Pixels: Exploring the Therapeutic Mechanisms of Virtual Nature for Young Adults’ Mental Well-Being
Source: Healthcare (Basel). 2025 Apr 14;13(8):895. doi: 10.3390/healthcare13080895 (PMC12027312; doi:10.3390/healthcare13080895)
Supplement: Supplementary file 1 [file healthcare-13-00895-s001.zip › Table S1.pdf]

Table S1. General information of interviewees (n = 35)

Participant No.	Gender	Age	Student	Educational Level	Work Type
P1	Male	24	Yes (Bachelor's)	Bachelor's degree	No
P2	Male	22	Yes (Bachelor's)	Bachelor's degree	Part time
P3	Female	22	Yes (Bachelor's)	Bachelor's degree	Part time
P4	Male	22	Yes (Bachelor's)	Bachelor's degree	Part time
P5	Male	22	Yes (Bachelor's)	Bachelor's degree	Part time
P6	Female	25	Yes (Bachelor's)	Bachelor's degree	Part time
P7	Female	27	No	Bachelor's degree	Full time
P8	Female	22	Yes (Bachelor's)	Bachelor's degree	Part time
P9	Female	22	Yes (Bachelor's)	Bachelor's degree	No
P10	Male	20	Yes (Bachelor's)	Bachelor's degree	Part time
P11	Female	21	Yes (Bachelor's)	Bachelor's degree	Part time
P12	Female	19	Yes (Bachelor's)	Bachelor's degree	No
P13	Female	22	Yes (Bachelor's)	Bachelor's degree	Part time
P14	Female	22	Yes (Bachelor's)	Bachelor's degree	Part time
P15	Female	24	Yes (Bachelor's)	Bachelor's degree	Part time
P16	Male	29	No	Bachelor's degree	Full time
P17	Female	24	Yes (Bachelor's)	Bachelor's degree	Part time
P18	Female	21	Yes (Bachelor's)	Bachelor's degree	No
P19	Female	21	Yes (Bachelor's)	Bachelor's degree	No
P20	Female	20	Yes (Bachelor's)	Bachelor's degree	Part time
P21	Female	22	Yes (Bachelor's)	Bachelor's degree	Part time
P22	Female	22	Yes (Bachelor's)	Bachelor's degree	Part time
P23	Female	23	Yes (Bachelor's)	Bachelor's degree	Part time

P24	Female	22	Yes (Bachelor's)	Bachelor's degree	Part time
P25	Male	23	Yes (Bachelor's)	Bachelor's degree	Part time
P26	Female	22	Yes (Bachelor's)	Bachelor's degree	Part time
P27	Female	22	Yes (Bachelor's)	Bachelor's degree	Part time
P28	Male	21	Yes (Bachelor's)	Bachelor's degree	Part time
P29	Male	22	Yes (Bachelor's)	Bachelor's degree	No
P30	Female	20	Yes (Bachelor's)	Bachelor's degree	Part time
P31	Female	22	Yes (Bachelor's)	Bachelor's degree	Part time
P32	Male	20	Yes (Bachelor's)	Bachelor's degree	No
P33	Female	22	Yes (Bachelor's)	Bachelor's degree	Part time
P34	Male	21	Yes (Bachelor's)	Bachelor's degree	Part time
P35	Female	21	Yes (Bachelor's)	Bachelor's degree	Part time
