# Supplementary figures and images for: Finding Peace in Pixels: Exploring the Therapeutic Mechanisms of Virtual Nature for Young Adults’ Mental Well-Being
Source: Healthcare (Basel). 2025 Apr 14;13(8):895. doi: 10.3390/healthcare13080895 (PMC12027312; doi:10.3390/healthcare13080895)

**Supplementary Materials File S1.** Virtual natural environments Undersea

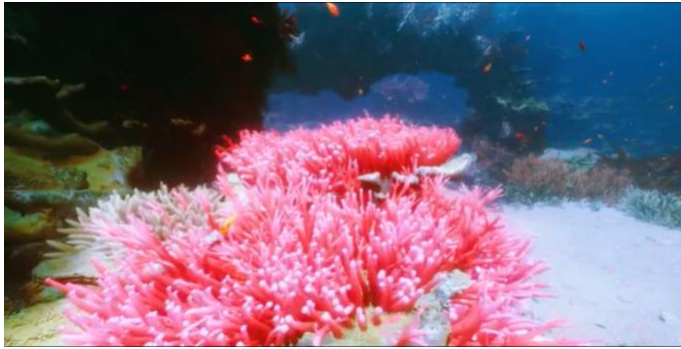

Sky

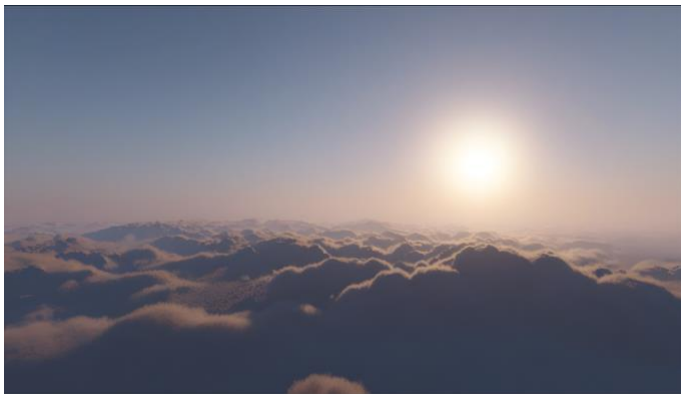

Forest

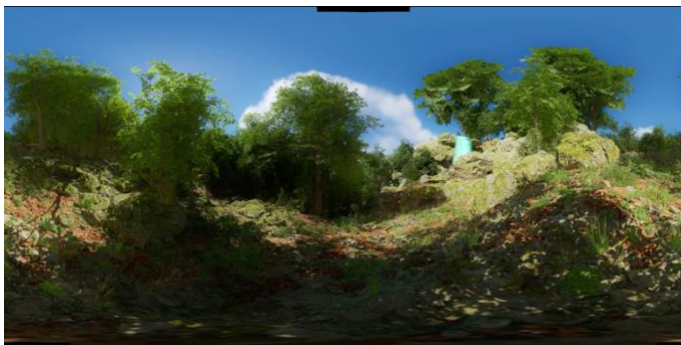

River

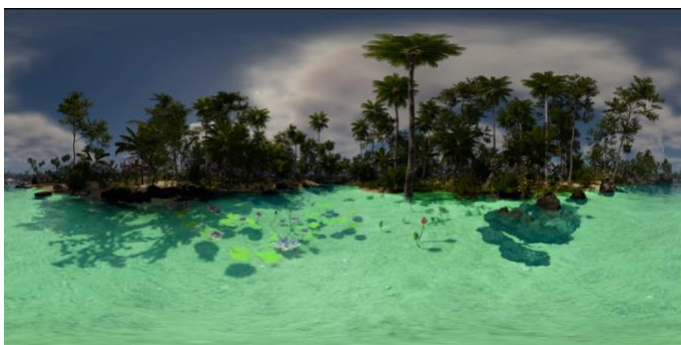

Supplement: Supplementary file 1 [file healthcare-13-00895-s001.zip › Supplementary Materials File S1. Virtual natural environments.pdf]
